# Vestibulo-spatial navigation: pathways and sense of direction

**DOI:** 10.1152/jn.00422.2022

**Published:** 2023-02-08

**Authors:** Athena Zachou, Adolfo M. Bronstein

**Affiliations:** Neuro-otology Unit, Department of Brain Sciences, Imperial College London, Charing Cross Hospital Campus, London, United Kingdom; 1st Department of Neurology, Eginition Hospital, National and Kapodistrian University of Athens, Greece

**Keywords:** downbeat nystagmus, GPS, sense of direction (SOD), vestibular navigation

## Abstract

Aims of the present article are: *1*) assessing vestibular contribution to spatial navigation, *2*) exploring how age, global positioning systems (GPS) use, and vestibular navigation contribute to subjective sense of direction (SOD), *3*) evaluating vestibular navigation in patients with lesions of the vestibular-cerebellum (patients with downbeat nystagmus, DBN) that could inform on the signals carried by vestibulo-cerebellar-cortical pathways. We applied two navigation tasks on a rotating chair in the dark: return-to-start (RTS), where subjects drive the chair back to the origin after discrete angular displacement stimuli (path reversal), and complete-the-circle (CTC) where subjects drive the chair on, all the way round to origin (path completion). We examined 24 normal controls (20–83 yr), five patients with DBN (62–77 yr) and, as proof of principle, two patients with early dementia (84 and 76 yr). We found a relationship between SOD, assessed by Santa Barbara Sense of Direction Scale, and subject’s age (positive), GPS use (negative), and CTC-vestibular-navigation-task (positive). Age-related decline in vestibular navigation was observed with the RTS task but not with the complex CTC task. Vestibular navigation was normal in patients with vestibulo-cerebellar dysfunction but abnormal, particularly CTC, in the demented patients. We conclude that vestibular navigation skills contribute to the build-up of our SOD. Unexpectedly, perceived SOD in the elderly is not inferior, possibly explained by increased GPS use by the young. Preserved vestibular navigation in cerebellar patients suggests that ascending vestibular-cerebellar projections carry velocity (not position) signals. The abnormalities in the cognitively impaired patients suggest that their vestibulo-spatial navigation is disrupted.

**NEW & NOTEWORTHY** Our subjective sense-of-direction is influenced by how good we are at spatial navigation using vestibular cues. Global positioning systems (GPS) may inhibit sense of direction. Increased use of GPS by the young may explain why the elderly’s sense of direction is not worse than the young’s. Patients with vestibulo-cerebellar dysfunction (downbeat nystagmus syndrome) display normal vestibular navigation, suggesting that ascending vestibulo-cerebellar-cortical pathways carry velocity rather than position signals. Pilot data indicate that dementia disrupts vestibular navigation.

## Graphical abstract

**Figure:**
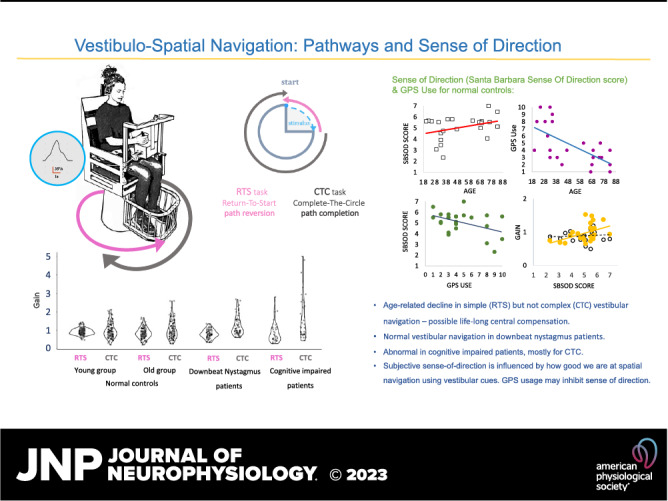

## INTRODUCTION

Spatial navigation in birds and fish involves three spatial dimensions but in humans it is largely confined to the horizontal plane. An additional simplification in human navigation is that, under normal illumination conditions, spatial navigation is mostly mediated by the visual system (1). In the dark, however, we rely on vestibular, proprioceptive, and motor reafference information to orient ourselves—critical for blind patients (2–4). Walking to the toilet at night with the lights off, or finding our tent when camping on a dark night are common but challenging examples. Thus, two aims of this study are, identifying a vestibular contribution to spatial navigation and how this may transfer to our subjective sense of direction (SOD), that is, our ability to find our way around and to know in which direction surrounding objects are.

Postural and ocular effects of lesions in the vestibular system can be compensated for by visual and proprioceptive inputs through remarkable processes of neural plasticity (5). Some aspects of vestibular failure however can never be fully compensated because the semicircular canals are the only sensory receptors specialized in sensing head rotation. Controlled experiments in blindfolded, well-compensated patients lacking vestibular function show considerable errors during walking triangle completion tasks (3). The distance walked (linear translation) is accurate but, failing to sense rotations, significant angular errors accumulate in patients. Experiments on a rotating chair in the dark show that angular spatial orientation is permanently lost in bilateral labyrinthine defective subjects (6) but in partial lesions performance also depends on central compensation processes (7). Unilateral vestibular lesions have been known to cause navigation bias when walking for over a hundred years, as examined by the star-shaped gait deviation on the Babinski-Weill test (8).

Experimental work indicates that the vestibular system is important for spatial navigation and orientation (9, 10). However, given that vestibular path integration is error prone and necessitates visual, proprioceptive, and efference copy input (11–13), whether vestibular cues are utilized for building up a SOD in healthy humans still is a source of debate. Most people are happy to define themselves as having a good or poor SOD but a quantitative assessment of our perceived SOD has been facilitated by questionnaires, chiefly the Santa Barbara SOD questionnaire (SBSOD) (14). This questionnaire shows good correlations with various spatial navigation/orientation tasks (15). Some of these navigation tasks used field experiments involving real self-motion (14, 16) but most used pen-and-paper or computer-based tests, hence without actual self-motion or vestibular activation (17). Results are therefore contradictory, some studies concluding that vestibulo-proprioceptive cues do (14) but some that they do not (16) contribute to our sense of direction. To the best of our knowledge, however, no study has attempted to selectively measure the vestibular contribution to SOD; this is another aim of this study using a technique exclusively targeting the horizontal semicircular canals. On the assumption that the assessment of our own SOD is based on a lifetime average of experiences under different sensory conditions (e.g., light vs. dark, walking vs. vehicular transportation, coherent vs. conflictive visuo-vestibular interaction), the contribution of an inertially based system such as the vestibular system should represent a robust input to the building up of our SOD. The prediction is that better performance during vestibular navigation tasks should be associated with a better SOD.

Age and habits also influence navigation and a sense of direction. A recent article entitled “Habitual use of GPS negatively impacts spatial memory during self-guided navigation” indicated that SOD was downmodulated by global positioning systems (GPS) use, but the research only involved young subjects (18). As the use of online technology is adopted more readily by the younger generations, any GPS-induced interference with spatial abilities may be reflected differently across the lifespan. In particular, it could be predicted that if the elderly use GPS less than the young, their spatial abilities and SOD could be relatively spared. However, as age may also be associated with reductions in spatial performance (19), the interaction between these two variables is difficult to predict. Hence, another aim of this study is to see how age, GPS usage, and vestibular navigation impact SOD.

As to how vestibular signals reach higher levels in the central nervous system (CNS) to generate conscious perceptual and orientational responses very little information is available in humans (9). An influential review (20) provides a conceptual summary. One of these ascending pathways, the vestibulo-cerebellar pathway, synapses in the vestibulo-cerebellum (the archi-cerebellum: floculo-nodular lobe and uvula) and reaches the hippocampus through the ventral lateral nucleus of the thalamus (VLN). However, what information is encoded in this pathway is unknown. In a previous study (21), we postulated that this pathway may be carrying vestibular head velocity (rather than position) signals, on the basis that patients with vestibulo-cerebellar lesion (downbeat nystagmus syndrome, DBN) showed reduced angular velocity self-motion perception, namely, a shortening of 50% of vestibular-perceptual time constants during constant angular velocity steps. [Nb: Human and nonhuman primate research shows that DBN is due to lesions of the vestibulo-cerebellum, in particular the floculo-nodular lobe (22, 23)]. In addition, the cerebellum has been suggested to be directly involved in spatial navigation given that the head-to-world reference frame conversion of vestibular signals may be taking place in lobules IX and X (24–26). Here, we investigate vestibular navigation in patients with DBN. We postulate that if patients with DBN showed defective vestibular navigation in response to angular displacement stimuli within the optimal vestibular frequency range (i.e., avoiding low frequencies), this would indicate that, in addition to head velocity signals, vestibulo-cerebellar projections also carry position/orientation signals.

Finally, although clinical evidence indicates that the right temporo-parietal junction plays a major role in human vestibularly mediated spatial orientation (27), the literature points at the hippocampus and rhinal cortices as major centers mediating spatial orientation and navigation (28–31). Hence, as proof of principle, we piloted vestibular navigation in two patients with early dementia [one with Alzheimer’s disease (AD) and one with Parkinson-plus syndrome, both presenting with spatial disorientation], as those brain areas are affected early in dementia. We deliberately chose two demented patients with different underlying pathology because one of the aims of this pilot study is to investigate if any dementia could potentially disrupt vestibular navigation.

In summary, the interrelated research questions addressed in this study are *1*) whether vestibular navigation makes a contribution to our subjective SOD; *2*) how GPS usage, age, and vestibular navigation skills interact in the build-up of our SOD; *3*) we assess patients with downbeat nystagmus to see whether the vestibulo-cerebellum is involved in vestibular spatial navigation. Finally, we carried out a pilot study in two patients with early dementia and spatial disorientation, to see if vestibular navigation is compromised. The vestibular navigation tasks use a motorized rotating chair and have been reported elsewhere (2) but essentially involve discrete rotational displacement stimuli followed by either a simple task requiring subjects to return to the starting position (path reversal) or a more integrative task requiring subjects to continue along to find the start completing the circle (path completion).

## MATERIALS AND METHODS

### Experimental Apparatus

**Figure 1.**
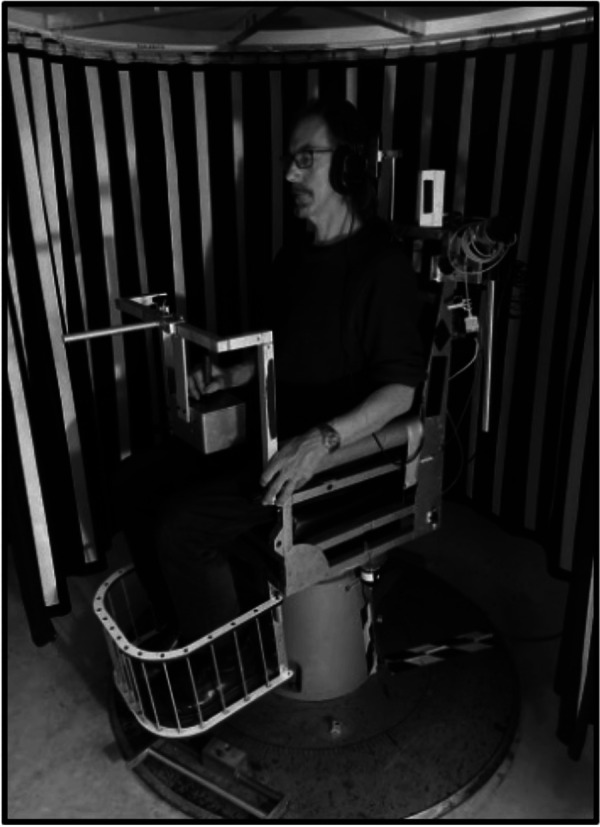
Apparatus used for the self-rotation test. Chair self-rotation (left or right) implemented by a directionally congruent joystick. The subject is seen holding the joystick with his right hand.

Details of the apparatus and procedures of the vestibular navigation task have been fully presented elsewhere (2). In summary, subjects were seated on a motorized, computer-driven, vibration-free rotating chair (Contravez DC motor; torque 120 Nm) surrounded by an optokinetic drum in total darkness (Fig. 1). White noise was delivered via headphones to mask auditory cues potentially coming from the laboratory. A padded, rigid occipital head rest was used to avoid head movements during rotations. Subjects were continuously observed via an infrared camera. The chair tachometer (velocity) and angular displacement (position output) were recorded at 250 Hz onto the computer with a National Instruments ADC card PCI-6025E. Chair displacement and movement duration was obtained from a position encoder.

The starting position was defined by means of a visual target attached to the striped curtain at 90 cm (drum radius) from the subject’s eyes, visualized by the subjects before each experimental run. The visual target was also used to update the subject’s orientation during lights-on breaks. Subjects could actively rotate themselves using a directionally congruent joystick attached to the chair, with a maximum angular velocity limited to 65°/s.

### Experimental Protocol

#### Vestibular navigation tasks.

The aim of the experiments was to quantify the subjects’ ability to reach the angular position where they were at just before a discrete rotational stimulus was applied (the starting position). Stimuli and responses took place in the dark. Before each of these runs the starting position was shown in the light for several seconds, visually reinforced by the presence of a large target attached to the inside of the rotating drum, straight ahead of the subjects. The target was an A4-size photograph of the laboratory, exactly as the subjects had seen it before sitting on the chair. This helped to visually remind subjects that they were in a broader environment, not just within an optokinetic drum.

**Figure 2.**
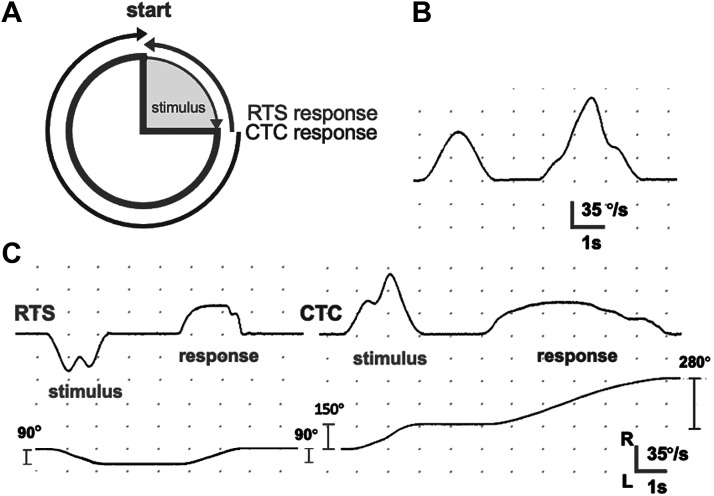
*A*: plan view showing a schematic of the tasks required by subjects in return-to-start (RTS) and complete-the-circle (CTC). *B*: typical recorded “smooth” and “noisy” stimuli chair velocity traces. *C*: velocity (*top*) and position (*bottom*) traces for an accurate RTS and a hypermetric CTC response.

Subjects were passively rotated in the dark and with their eyes closed through eight different amplitudes (60°, 90°, 120°, 150°, 210°, 240°, 270°, and 300°). The duration of each stimulus was fixed (duration range 1.72–3.85 s) and frequency content was within the optimal vestibular frequency range (0.26–0.58 Hz). Subjects were always instructed to return to the starting position, either by going backward (return-to-start, RTS), or forward and completing the circle (complete-the-circle, CTC) (Fig. 2, *A* and *C*). Small runs of 5–10 RTS or CTC responses were studied; the direction (right or left), the order of RTS and CTC runs as well as the stimuli amplitude order was pseudorandomized. Subjects were instructed to wait for ∼2 s before they responded to each stimulus to minimize any postrotational effect.

A pilot study in normal subjects (*n* = 19, age range 22–83 yr) aimed to determine if irregular stimuli combining several frequencies (“noisy stimuli”) would induce worse performance than single frequency stimuli (“smooth stimuli”) (Fig. 2*B*). The latter consisted of raised cosine angular velocities profiles ranging in peak velocities from 55 to 128°/s, with a peak acceleration of 100°/s^2^. The former (“noisy”) stimuli combined additional sinewaves of 0.25, 0.7, and 1.3 Hz (peak angular velocity 66–140°/s, peak acceleration 100°/s^2^) superimposed onto the fundamental single frequency (range 0.26–0.58 Hz). Added “noise” did not affect the stimulus’ duration. Despite the fact that the pilot study did not show differences between “smooth” and “noisy” stimuli (see results), for the main study we decided to use only “noisy” stimuli because we assumed that subjects would find it more difficult to respond by just copying the dynamic profile of the preceding stimulus (recall that we are assessing the person’s ability to compute angular position, not reproducing velocity profiles). All stimuli were created using custom-made software (ARbwave by D. Buckwell, Imperial College London).

In summary, the experimental design forced subjects to only use a position strategy during their response, namely, *1*) the velocity waveform of the stimuli was too complex to be resolved, *2*) CTC task can only be accomplished by computing the stimulus and response in displacement units, *3*) the velocity output of the joystick was deliberately curtailed so that, even if subjects tried, they would not be able to simply reproduce the dynamic profile of the stimuli.

During the practice session, participants received randomized stimuli for both RTS and CTC tasks, with lights on and eyes open and with eyes closed and lights off. Subjects proceeded to the experiment once the experimenter was reassured that they understood and could perform the two tasks.

Participants received 30–40 noisy stimuli in total, interleaved in a balanced order of presentation design, between RTS and CTC. The interval between trials was 2–5 s. Subjects were brought back to the starting position, if needed, by the experimenter every 5–10 trials, just before each break. To achieve that, in darkness with eyes closed, the position of the optokinetic drum was adjusted in case there was any drift so that participants would always face the starting position on opening their eyes. This precluded participants receiving any feedback of their errors. During the break, participants opened their eyes and reorientated themselves by looking at the visual target, always in front of them.

Responses were considered “wrong” and rejected when subjects would perform an RTS response instead of a CTC and vice versa; when it would be the right task but in the wrong direction (i.e., going backward to complete the circle) or when there was no response at all. Also, responses with frequency < 0.10 Hz (=lasting > 10 s) were eliminated. The gain was used as the measurement of performance which was defined as the ratio of the actual response to the ideal response, both measured in degrees: Gain = Actual Response/Ideal Response.

### Normal Subjects

We tested 24 healthy controls with no history of neurological or vestibular disease (age range 20–83 yr; mean age = 51.4, 9 women). For age-related comparisons, controls were clustered into two groups: Young (20–35 yr; *n* = 10; mean age =29.6, SD = 5.4) and Old (60–83 yr; *n* = 11; mean age = 73.5, SD = 7.4). None of the old healthy controls had presbyvestibulopathy [according to the inclusion criteria for presbyvestibulopathy in the study by Agrawal et al. (32)] due to the absence of vestibular symptoms and normal vestibulo-ocular reflex gains [Lateral canal video head impulse test (vHIT) mean gain = 1.03, SD = 0.07; range = 0.85–1.19, bilaterally] (32). Three middle-age healthy control subjects were added for age-related correlational analysis (aged 39, 43, and 47 yr). No vHIT measurements were undertaken in young- and middle-aged healthy subjects.

### Patients

Five patients with primary gaze spontaneous downbeat nystagmus (DBN), gait unsteadiness but minimal or no limb ataxia (age range 62–77, mean age = 70.4, SD = 5.9, 3 women) participated in the study. All cases had the diagnosis of midline cerebellar syndrome of unknown etiology with no neurological family history, or idiopathic DBN syndrome, for short (33). The nystagmus was seen clinically in all cases and enhanced on ocular convergence, lateral gaze, head shaking, and positioning, as customary in this syndrome (34). Brain MRI scans were reported as superior cerebellar atrophy in one patient and flocculo-nodular atrophy in a second patient, whereas the remaining three were considered normal. DBN is reproduced in monkeys by flocculectomy (22); in agreement, functional imaging shows abnormal vestibulo-cerebellar activity (23).

**Figure 3.**
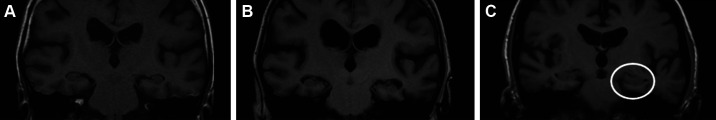
Coronal T1 brain MRI scans for *D1* patient at the age of 78 yr (normal) (*A*), same patient (*D1*) (*B*), 6 years after the first MRI scan, showing significant hippocampal atrophy, and normal control of similar age (83 yr old; left hippocampus in white circle) (*C*).

We also tested two patients with neuro-degenerative cognitive impairment (CI) as a feasibility study on the effect of dementia on vestibular navigation. *Patient D1* was an 84-yr-old woman who complained of nonspecific dizziness and spatial disorientation episodes. Her daughter volunteered that she needed help in everyday life activities and housework. She had been MRI scanned just before consultation showing a significant reduction of hippocampal volume (Fig. 3*B*). Neurological examination revealed short-term memory impairment but normal sensorimotor, vestibular, and eye movement examination. She had a 17/30 MOCA score (35, 36). A clinical diagnosis of probable early AD was made (37, 38). She had a previous MRI scan six years earlier, for headaches that resolved spontaneously, which was normal (Fig. 3*A*).

*Patient D2* was a 76-yr-old man with a 3-yr sense of vague imbalance and a fall. Examination at that point was normal and brain and neck MRI was unremarkable. On a recent follow-up, he complained of worsening balance, falls, memory loss, and episodes of spatial disorientation (struggled to find a familiar shop at a known shopping mall). He experienced vivid and acted-out dreams. On examination, he had postural blood pressure drops, moderate gait ataxia, a tendency to fall during retropulsion test, and marginal DBN on lateral gaze and supine. A new MRI scan reported a moderate but generalized reduction in brain volume since the previous scan. A DAT scan showed reduced dopamine transport in the putamen and caudate nuclei. Although a diagnosis of early Alzheimer’s disease/mild cognitive impairment was produced by his local memory service, at our institution he was thought to suffer from either multiple system atrophy with cognitive impairment or early Lewy body disease dementia (39).

All patients had been recruited through our neuro-otology clinic (AMB) and had normal peripheral vestibular function in the yaw plane, clinically and on at least one of the following laboratory tests: video-head impulse test, caloric or rotational chair testing. Vertical oculography, however, was not undertaken due to the large variability present for this parameter in patients with DBN (40–43).

All procedures were conducted in accordance with the Declaration of Helsinki. All procedures were also approved by the local ethics committee and all participants provided written informed consent.

### Questionnaires and GPS Use

The Santa Barbara sense of direction (SBSOD) scale was used to quantify subjects’ self-reported spatial ability (14). SBSOD scale is a self-reported measure of spatial orientation consisting of 15 questions and a scale to quantify responses from 1 to 7. Representative questions are: “I am very good at judging distances”; “I tend to think of my environment in terms of cardinal directions (N, S, E, W)”; “I can usually remember a new route after I have traveled it only once.” Total scores were calculated by adding the score of each question and then divided by the number of questions. Higher scores indicate a better subjective sense of direction. We wanted to explore if SOD in normal people is affected by the use of GPS navigation applications. Hence, GPS exposure was quantified using a 1–10 ranked scale, where 1 corresponds to “I never use GPS” and 10 “I use GPS constantly, everywhere.” Subjects were asked to evaluate the use of GPS during the preceding month including all everyday activities, walking, commuting, and driving. A MOCA cognitive test was obtained for all healthy participants older than 60 yr old which showed they were all within the normal range (26–30/30).

### Statistical Analysis

Responses with frequency < 0.10 Hz were eliminated before any statistical analysis: in total 26/676 responses in normal controls, 11/173 in patients with DBN, and 3/75 in cognitively impaired patients. For the pilot study looking for performance differences between “smooth” and “noisy” stimuli in healthy controls repeated-measures ANOVA was used. To evaluate the relationship between age and task performance as well as between stimuli amplitude and task performance, we used Spearman’s correlation coefficients. For age-related comparisons, normal controls were grouped into two groups (“Young” and “Old”), as reported earlier. To explore the interaction between age, task, and amplitude, we used a three-way mixed model ANOVA with one between-subjects factor: age group (Young-Old) and two within-subjects factors: task (RTS-CTC) and stimulus amplitude (60°–300°). For the comparison of normal subjects with a small patient group (patients with DBN, *n* = 5), we applied nonparametric statistics. Data are expressed as median and interquartile range (IQR) when using nonparametric presentation; means and SD was used to express data in that case. For each case, data points lower than Q1 − 1.5 ×  IQR (where Q1 is the first quartile), or higher than Q3 + 1.5 ×  IQR (where Q3 is the third quartile), or >2.5 SD were eliminated as outliers. Wilcoxon signed ranks test was used to further explore differences between the two vestibular navigation tasks. To investigate how variables such as gain responses for both RTS and CTC tasks, sense of direction scores, GPS exposure, and age relate to each other, we used Pearson’s bivariate correlations and to explore how these correlations may group themselves we used factor analysis (extraction method: principal component analysis, rotation method: varimax with Kaiser normalization). Significance was defined as an α level of 0.05 or less. All statistical analysis was conducted in SPSS Statistics 26 (IBM Corp.).

## RESULTS

We conducted a pilot study to identify if introducing additional sinewaves (“noise”) to the stimuli affected performance in the vestibular navigation tasks. We compared gain responses between smooth and noisy stimuli in healthy controls and we found no differences in performance in any task (*n* = 19, *F* = 0.351, *P* = 0.56, repeated-measures ANOVA). As in principle it would be safer to use the noisy stimulus to identify navigational (positional) behavior, all data reported here relate to these stimuli.

### Age and Amplitude Effects on Vestibular Navigation

**Figure 4.**
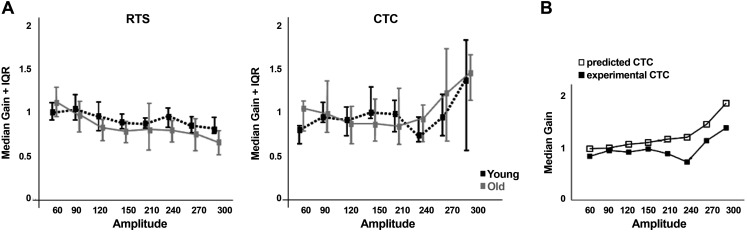
*A*: median gain and interquartile range (IQR) of responses at eight stimuli amplitudes for Young (*n* = 10, 20–35 years, 7 women; dark dotted line) and Old groups (*n* = 11, 60–83 yr, 2 women; solid gray line) for RTS (return-to-start) and CTC (complete-the-circle) tasks. Old subjects show a small but consistent lower response gain in RTS [Interaction between age group and task: *F*(1,19) = 6.137, *P* = 0.02, three-way mixed ANOVA]. *B:* predicted and experimental CTC: median gain values per amplitude for all normal controls (*n* = 24, age range 20–83 yr, 9 women). Experimental CTC (filled squares) are hovering closer to gain = 1 than predicted CTC gain (open boxes), (*z* = −3.727, *P* < 0.001, Wilcoxon signed-rank test).

Figure 4*A* shows the median gain and the interquartile range (IQR) of responses for the young and old normal subjects at different stimuli amplitudes. In general, responses are accurate, hovering around unity gain. There is modulation of the response by stimulus amplitude, shown as a tendency for gains <1 during “return to start” (RTS) task and >1 during “complete the circle” (CTC) task, for the larger stimulus amplitudes. A three-way mixed model ANOVA confirmed a significant task effect [*F*(1,19) = 14.146, *P* = 0.001] but no significant amplitude effect [*F*(7,133) = 0.944, *P* = 0.48]. The interaction between task and amplitude though was significant [*F*(7,133) = 2.409, *P* = 0.02) and, in agreement, correlation analysis showed a negative correlation between RTS task and amplitude (*r* = **−**0.34, *P* < 0.01) but only a trend (positive) for CTC (*r* = 0.10, *P* = 0.07). This amplitude effect is likely due to the reduced sensitivity of the semicircular canals for lower frequency stimuli. Despite the fact that the frequency content of our stimuli (0.26–0.58 Hz) is within the frequency range of the vestibular system, full gain responsiveness across species is achieved at frequencies of 0.2 Hz and upward (44, 45).

Figure 4*A* also suggests that the subjects’ age affects performance differentially. Accordingly, although main effect for age group was not significant [*F*(1,19) = 1.306, *P* = 0.27], the interaction between age group and task was [*F*(1,19) = 6.137, *P* = 0.02, three-way mixed ANOVA]. Additional correlational analysis, including the three middle-aged subjects, showed a low but significant negative correlation between age and RTS performance (*r* = –0.29, *P* < 0.001) but, again, no correlation for CTC (*r* = −0.07, *P* = 0.26). The aforementioned results were confirmed by nonparametrical analysis.

Although Fig. 4*A* suggested a different age-dependent effect of amplitude on RTS-gain (= interaction between age group and amplitude), this was not confirmed statistically (no interaction between age group and amplitude was found [*F*(7,133) = 1.874, *P* = 0.12, three-way mixed model ANOVA].

Finally, the Old group showed greater variability than Young controls in both tasks; both groups had higher variance in CTC responses [top of Table 1; main effect for task: *F*(1,19) = 23.268, *P* < 0.001; main effect for age group: *F*(1,19) = 5.279, *P* = 0.03; no significant interaction between task and age: *F*(1,19) = 3.735, *P* = 0.07, two-way mixed design ANOVA within task and between age group].

**Table 1.** Variability of median gain responses

Group	RTS	CTC
Young controls	0.27	0.57
Old controls	0.45	0.70
DBN patients	0.43	0.68
CI patients	0.90	1.50

Variability expressed as the ratio of interquartile range to the median gain (IQR/median gain) for control groups, downbeat nystagmus (DBN), and cognitive impaired (CI) patients, for both return-to-start (RTS) and complete-the-circle (CTC) tasks.

Due to RTS-CTC discrepancies mentioned earlier, we explored the comparative performance between the two tasks. First, we found no correlation between RTS and CTC gains in normal controls (Pearson’s correlation coefficient *r* = 0.23, *P* = 0.29). To further confirm that RTS and CTC induce different responses, we explored the following assumption: the setup of the experiment forces the subjects to only rely on their rotational perception to be able to perform the tasks. If the perceived angle in RTS for one stimulus is *x* degrees, then in CTC the response will be 360° − *x* for the same stimulus. In that case, we can predict CTC gain using RTS gain and explore the hypothesis that predicted CTC would not be different from experimental CTC. Accordingly, we compared predicted-CTC (from RTS gain values) with the experimental CTC gain values. We calculated the mean predicted-CTC and experimental-CTC gain per amplitude for each subject. First, we compare for all normal controls and then we explore the differences for each age group separately.

A paired analysis showed that predicted CTC was significantly higher than experimental CTC gain values for all normal controls (*z* = −3.727, *P* < 0.001; median gain was 1.04 and 0.94, respectively) and for the two age groups separately (Young: *z* = −3.891, *P* < 0.001; Old: *z* = −2.750, *P* = 0.006, Wilcoxon signed-rank test). These results showed that experimental CTC responses were different and even better (more veridical) than our hypothesis predicted (Fig. 4*B*).

### Vestibular Navigation, Sense of Direction, and GPS Use

**Figure 5.**
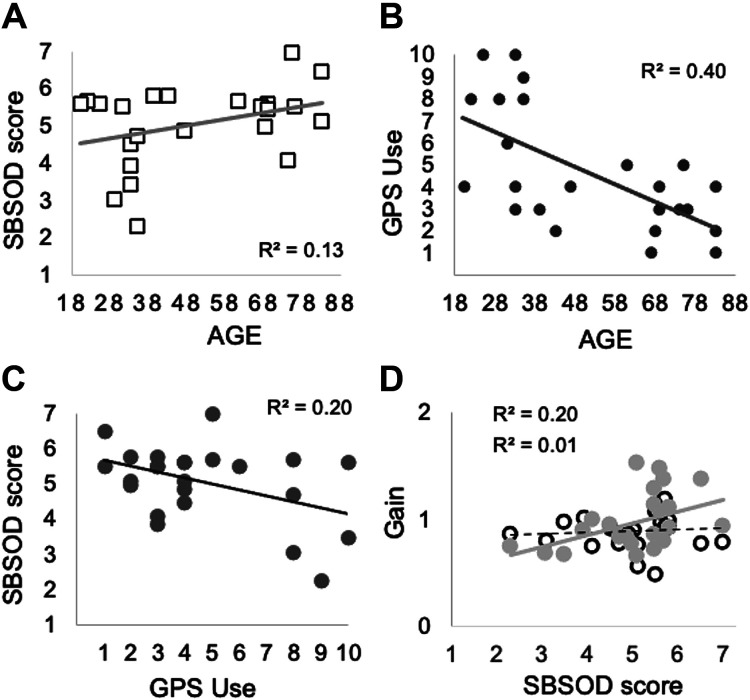
Visual representation of bivariate correlations: *A*: effect of age on the Santa Barbara Sense of Direction Scale scores (SBSODs) across normal subjects (*n* = 24, 20–83 yr, 9 women); SBSOD scores show a trend to increase with age (*r*^2^ = 0.13; *P* = 0.08). *B:* the relationship between age and GPS exposure (GPS use) across all normal subjects. As expected, GPS use diminishes with age (*r*^2^ = 0.40; *P* < 0. 01). *C*: the relationship between GPS exposure and scores on SBSOD; subjects using GPS systems more show lower SBSOD scores (*r*^2^ = 0.20; *P* = 0.03). *D*: SBSOD scores vs. vestibular navigation tasks [return-to-start (RTS), open circles, and complete-the-circle (CTC), filled circles; each dot represents a median gain value for all eight amplitudes per task and healthy subject. Gain is defined as the ratio of actual response to the ideal response, as described in materials and methods. The rationale for plotting these data is that sense of direction might be partly mediated by good vestibular navigation skills. Performance in the simpler RTS task is not associated with SBSODs (*r*^2^ = 0.01; *P* = 0.72) whereas performance during the more complex CTC task shows a small association with SBSODs (*r*^2^ = 0.20; *P* = 0.03). Re-running this correlation excluding the five subjects with CTC gains above unity gain does not essentially change this relationship (*r* = 0.43; *P* = 0.07). When Bonferroni corrections were adjusted, only correlation between age and GPS use remained statistically significant.

To gain insight on how these variables (Fig. 5) associate with each other to modulate sense of direction, we carried out factor analysis (FA). Factor analysis extracted two components explaining 71% of the total variance in the data. Table 2 shows that with a standard cut off point of 0.4 for factor loading-in, *component 1* (41% of data variance) loads SBSOD scores, participants’ age (positively), and GPS use (negatively). *Component 2* (30% of data variance) also loads the SBSOD scores and both vestibular navigation task results (positively). Loading values for age, GPS use, and CTC gain were all pretty high, above 0.8.

**Table 2.** Factor analysis results

Variables	*Component 1*	*Component 2*
SBSOD score (1–7)	0.625	0.584
Age	0.883	−0.242
GPS Use (1–10)	−0.837	−0.130
RTS median Gain	−0.389	0.673
CTC median Gain	0.147	0.813

Component matrix with varimax rotation: two components were extracted for the interaction of the five variables. *Component 1* loaded significantly Santa Barbara Sense of Direction Scale (SBSOD) score, age, and GPS use and *component 2* loaded SBSOD score and both return-to-start (RTS) and complete-the-circle (CTC) tasks.

### Vestibular Navigation in Neurological Patients

**Figure 6.**
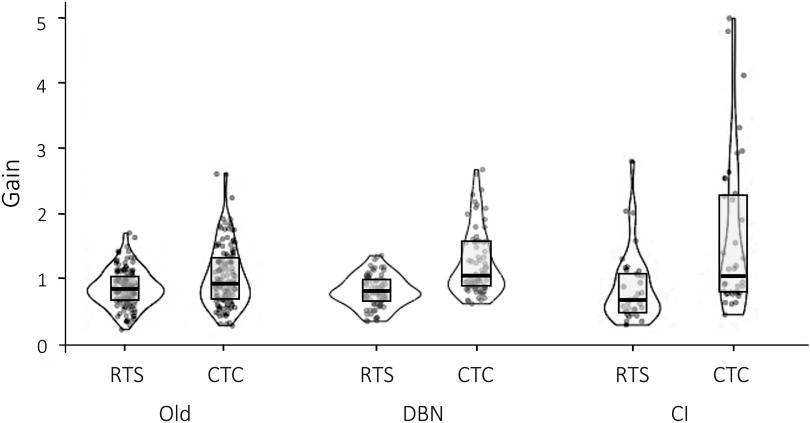
Violin plots (46) for Old healthy subjects (*n* = 11, 60–83 yr, 2 women), patients with downbeat nystagmus (DBN) (*n* = 5, 62–77 yr, 3 women) and the two patients with cognitive impairment (CI) (a woman 84 yr old and a man 76 yr old). They include all individual data points for each group and task [Old return-to-start (RTS) *n* = 131, Old complete-the-circle (CTC) *n* =129; DBN RTS *n* = 80, DBN CTC *n* = 72; CI RTS *n* = 31, CI CTC *n* = 39). Light gray boxes represent interquartile range (IQR) range and the thick black line median gain.

Direct observation of the patients with DBN during the experiment confirmed that they understood the vestibular navigation tasks well and were accurate with their responses. Essentially, patients with DBN did not show abnormalities in the vestibular navigation tasks. Figure 6 shows individual gain responses for the five patients with DBN (age range = 62–77) and 11 old healthy subjects of a similar age (age range = 60–83). The median gain of the individual responses for patients with DBN was 0.79 (IQR 0.34) for RTS and 1.04 (IQR 0.71) for CTC; for the elderly control group, median gain was 0.84 (IQR .38) for RTS and for CTC 0.93 (IQR 0.65). There were no control-patient group differences in any task (RTS: *U* = 27.0, *Z* = −0.057, *P* = 0.95; CTC: *U* = 15.5, *Z* = −1.36, *P* = 0.18, Mann–Whitney *U* test).

Patients with DBN had a slightly reduced mean SBSOD score (mean SBSODs = 4.13, SD = 0.98, *n* = 5) than the age-matched controls (mean SBSODs = 5.51, SD = 0.76, *n* = 11; *U* = 9.500, *Z* = −2.048, *P* = 0.04, Mann–Whitney test). This could be due to the nystagmus-induced visual instability (oscillopsia) interfering with navigation in the real world, which is what the SBSOD questionnaire quantifies.

Direct observation of the two cognitively impaired patients during the experiment showed they had difficulties executing the tasks, requiring more time and repetitions to grasp them, more practice time, and more breaks. As they got tired quicker, they were unable to perform as many trials as the controls did. Figure 6 shows that these two patients had less accurate and very variable performance compared with elderly normal controls. This was seen in both tasks but particularly CTC where they made frequent and large overestimation errors; the two cognitive patients had gains above 2.0 in 32% of CTC-responses (*n* = 39) but this was seen in only 3% of the 11 elderly controls (*n* = 129). They also executed more “wrong” responses (direction or task): 16% of all responses were wrong (RTS and CTC *n* = 70) whereas Old controls had only 1.8% of wrong responses (RTS and CTC *n* = 260). Finally, these patients showed greater variability than Old controls, especially in CTC task (Table 1).

## DISCUSSION

Spatial navigation and orientation are processes mediated by the hippocampus (47), para-hippocampal regions (29, 48–50), and the right temporo-parietal cortex (27), based on inputs from the visual, proprioceptive, and vestibular systems. In this article, we focus on how selective vestibular navigation contributes to our sense of direction, a topic largely neglected possibly due to the rotational techniques needed. A general limitation with spatial orientation research is that many standard tasks used involve participants seated motionless, e.g., the virtual Morris water maze procedures—totally neglecting the role of the vestibular system. When ecological tasks have been used (51–55), making inferences about specific sensory contributions can be difficult (56). In the first section of discussion, we examine how age and habitual factors, such as the use of GPS, interact with vestibular navigation to build up a subjective sense of direction. In the second part, we examine patients with lesions of the vestibulo-cerebellum discussing the information conveyed by a putative vestibulo-cerebellar-hippocampal pathway.

### Age and Habitual Effects on Sense of Direction and Vestibular Navigation

#### The effect of age on vestibular navigation.

We adopted two self-rotation paradigms (2), one is the “Return to Start” (RTS) task where subjects go back to the starting position by reversing the direction of the preceding rotational stimulus with the joystick. The second task “Complete the Circle” (CTC) is more complex and requires absolute spatial calibration because subjects must reach the starting position by continuing to rotate in the same direction of the stimulus. An analogy for RTS is leaving your home, walking to a neighbor’s house three or four doors down your street, and coming back via the same route, backtracking your steps. An analogy for CTC is going to the neighbor’s house but coming back home by walking all the way round the block. As we wanted subjects to use an orientational (position-based) strategy in their responses, the velocity output of the chair during the subject-driven responses was clipped so they could not just reproduce “the feel,” or dynamic profile, of the stimulus. In any case, the CTC task can only be performed with a position-based strategy. Importantly, neither of these tasks can be performed by subjects devoid of vestibular function (2, 6).

Figure 4*A* shows the presence of interaction of age and amplitude with RTS and CTC tasks. During RTS both subject groups, young and old, show a gradually declining response gain as a function of stimulus amplitude. This result is expected, as larger amplitude stimuli take longer to be completed and thus approach the frequency resolution imposed by the high-pass characteristics of the semicircular canals (see stimulus characteristics under material and methods). In addition, RTS gain in the old group is slightly but consistently lower than in the young, likely explained by a slight decrease in peripheral vestibular function with age (57–59). Even when normal subjects without any vestibular symptoms are studied, as we did, there is a slight but statistically significant decay in head impulse test gain between the ages of 20 and 80 yr (60). We recorded vHIT gains in our elderly normal controls to make sure they did not have gains below 0.8 (32) but, unfortunately, not in our young subjects, which is a limitation of the study. However, we would like to point out that the self-rotation RTS task has been proven to be a validated test of vestibular function (6, 61, 62). So, although a direct comparison between RTS and vHIT across age could not be carried out we cannot find an alternative explanation and still believe that the small age-related effect observed in the RTS task is due to slight age-related decay in vestibular responses.

Performance during the CTC task, unlike that for RTS, does not differ between the age groups. In contrast to RTS, CTC gain increases for larger (>240°) stimuli and responses become hypermetric (gains >1). However, it should be pointed out that both the hypometricity observed during RTS and hypermetricity during CTC as a function of stimulus amplitude are likely the result of the same underlying phenomenon. Reduced perception of an angular stimulus (as it approaches frequency resolution limits) will result in a shorter response to go back to start (RTS) and it will induce the subject to continue for longer to complete the circle (CTC).

Other differences between RTS and CTC tasks are less likely related to the reduced peripheral vestibular sensitivity of the semicircular canals to low-frequency rotation but more to the increased complexity of the CTC task. One such example is the larger variability in the CTC task compared with that of RTS, likely related to the additional central computations required for the CTC task. Although both tasks are mediated by the process known as path integration (63), RTS can be viewed as path reversal whereas CTC represents path completion where subjects need to plan a new route to reach their destination. These statements on the different nature of the two tasks are also supported by the absence of a significant correlation between RTS and CTC gains and by the comparison between predicted and experimental CTC—in agreement with previous results indicating different strategies for the two tasks (2).

In this regard, it is worth noting that the age effect observed during RTS was not seen during CTC which, statistically speaking, could be related to the larger variance in the latter than in the former. However, it is worth considering other reasons why age may affect RTS and CTC differentially. One could postulate that, in parallel to the slow loss of peripheral labyrinthine function during the life span, the brain is gradually implementing central vestibular compensatory processes, thus making up for what has been gradually lost in the periphery. Surprising as this may sound, good evidence for central propping up of diminished peripheral sensory information has been reported for the elderly both in the central auditory (64) and vestibular systems (65). A similar phenomenon has been observed with an RTS protocol in patients with vestibular schwannoma who lose vestibular function very gradually while simultaneously developing efficient central vestibular compensation (62). Further evidence from our current data will be presented later. In conclusion, age affects vestibularly based simple path reversal performance differently to the more complex path completion task. Interestingly, the former experiences age-related effects more than the latter.

#### The effects of GPS, age, and vestibular navigation abilities on sense of direction.

Global positioning systems (GPS) have become everyday use and, reportedly, university students “with greater lifetime GPS experience have worse spatial memory during self-guided navigation” (18). In broad terms, our results are in agreement but we now investigated how aging, GPS use, and vestibular navigation modulate SOD. The bivariate correlations in Fig. 5 followed by factor analysis showed that SOD is associated with age (positively), GPS use (negatively) (Factor 1), and with both vestibular navigation tasks gains (positively) (Factor 2). Loading values for age, GPS use, and CTC gain were particularly high, above 0.8.

The finding that sense of direction in the elderly appeared not to decay and that the likely explanation is that they use GPS less than the young is tantalizing. Nevertheless, this is supported by the aforementioned report of a dose-related deleterious effect of GPS usage on spatial memory in young subjects (16). As a possible mechanism, one could argue that, during ecological (non-GPS aided) spatial navigation, visual and vestibular inputs are coherent and additive. In contrast spatial navigation while watching a GPS device puts vestibular and visual mechanisms in conflict—vestibulo-ocular and optokinetic inputs become subtractive and antagonistic [vestibulo-ocular reflex suppression (66–68)]. It is therefore possible that repetitive use of GPS while navigating (i.e., under repetitive VOR suppression conditions) might inhibit our sense of direction. An alternative and perhaps simpler explanation could be that people navigating the world with a GPS device would spend less time, and attentional resources, looking at their surroundings. This might also lead to a worse subjective sense of direction. This is a testable hypothesis in a longitudinal study.

We believe that our results demonstrate the contribution of the vestibular system to perceived SOD in healthy human beings. The fact that the more demanding CTC task loaded more strongly than RTS in the factor analysis indicates that being able to integrate a preceding vestibular stimulus in new route planning contributes to our sense of direction. The CTC task requires a central representation of circular space and, as CTC but not RTS is subnormal in congenitally blind people (2) it is likely that this central representation relies on reciprocal visuo-vestibular recalibration.

Apart from our current findings, not much data on a specific vestibular contribution to the sense of direction in humans is available. A previous study in the elderly found that bilaterally absent C-Vemps (cervical vestibular myogenic-evoked potentials) were associated with a poorer sense of direction (69). C-Vemps are thought to represent short-latency activation of the saccule, but we find it perplexing that long body axis linear acceleration receptors can contribute to a sense of direction in humans. One possibility is that absent C-Vemps in subjects with lower SOD is an artifact associated with diminished cooperation in subjects with subclinical cognitive impairment. (Testing of C-Vemps requires sustained voluntary contraction of neck muscles, and they are often absent in old normal people.) Alternatively, C-Vemps might be a marker of more relevant central vestibular processing such as that investigated directly here with a CTC task.

Our findings relied on the fact that we took considerable care in defining the frequency range of our stimuli (by experimental design) and responses (by rejection of responses longer than 10 s). Thus, they were appropriate to the physical characteristics of the sensory receptors. In contrast, a recent study (16) concluded that vestibular input did not contribute to path integration although the authors themselves believed that the task adopted may have been inappropriate to probe vestibular contributions. Subjects had to travel a circular route with radii of 1 to 3 m, either actively walking or passively on a wheelchair, thus putting the semicircular canals out of their physiological frequency range. Studies using ecological tasks have identified a role for vestibular and proprioceptive inputs but disentangling the relative contribution of each of these senses, say, during walking, is not possible (51–55, 70).

Controlled vestibular stimulation studies have shown that the semicircular canals output (a signal coding head angular velocity) is mathematically integrated by the brain to generate a useful orientation signal (position)—hence providing a simple path integration mechanism (71, 72). So, the significance of our finding is not that the vestibular input can be path integrated but rather that, despite the nonecological conditions we used (subjects strapped to a rotating chair in the dark), an association between vestibular navigation and subjective SOD was present at all. Interestingly, the stronger CTC results emphasize the contribution of a complex vestibular-cognitive system to SOD rather than just a simple path reversal mechanism. We would therefore expect an even higher level of association between vestibular navigation ability and sense of direction in the dark, which is not assessed with current questionnaire tools. Although clinicians often ask bilateral vestibular patients how they balance in the dark, sense of direction is neither specifically probed by doctors nor spontaneously reported by patients. This specific topic warrants further research.

### The Effects of Neurological Disease on Vestibular Navigation

A main result of this study is that midline cerebellar patients (DBN group) did not show abnormalities during vestibular navigation. Biological navigation uses a sense of position in space and path integration, whereby preceding movements of an organism are stored and a new position to be traveled to can be programmed. Both these functions require angular vestibular input (3). Hippocampal neurons conveying a sense of position and orientation, e.g., head direction cells (10, 73), are profoundly disrupted after labyrinthectomy. Humans deprived of vestibular function are unable to integrate the angular component of a previously traveled path (3) an expected finding if we remember that the semicircular canals are the only organs specialized in transducing angular motion of the body. Although the vestibular nerve encodes angular velocity, central integrative process makes both, head velocity and position signals, widely available through the neuraxis (74–76).

We tested patients with downbeat nystagmus (DBN), a typical vestibulo-cerebellar sign, partly to further delineate the clinical picture, e.g., do cerebellar patients experience vestibular navigational deficits? In addition, understanding vestibular perception in these patients could afford preliminary insight on vestibulo-cerebellar-cortical projections in man and on the nature of the information conveyed by these pathways. Although little information is available on vestibulo-cortical cognitive tracts in humans, several pathways in mammals have been hypothesized including some via the vestibulo-cerebellum (20). An ascending vestibulo-cerebellar-cortical pathway identified in primates carries head velocity signals (77) and the findings in patients with DBN are in agreement with this because, *1*) patients with DBN show shortened time constants of vestibulo-perceptual velocity during velocity steps (21) and *2*) preserved vestibular navigation as shown in the current study would be incompatible with a vestibulo-cerebellar-cortical pathway carrying position or orientation signals. If the vestibulo-cerebellum had already mathematically integrated the head velocity input conveyed by the vestibular nerve, to convert it into a head-direction cell signal, patients with DBN would not have been able to complete our tasks successfully. The shortening of rotational velocity time constants previously reported (21) might have affected very low-frequency stimuli but these were deliberately avoided as per our current protocol.

This conclusion is also supported by the clinical picture in these patients. A nystagmus present in primary gaze, such as DBN, represents a vestibulo-ocular velocity bias (Nb nystagmus develops because of an approximately constant velocity drift of the eyes in one direction, the slow component of nystagmus, then followed by fast phase refixations) (78). Alternatively, in view of the normal results in our patients, it could be argued that pathways affected by cerebellar disease leading to DBN do not carry vestibular signals. Although one paper based on modeling argues that some of the findings present in patients with DBN may be explained by damage to gaze-velocity sensitive Purkinje cells (79), such hypothesis could at most account for ocular-motor features only. DBN is accompanied by gait ataxia and increased sagittal postural sway (80, 81), as well as dizziness (82) and shortened vestibulo-perceptual time constants (21), which are best explained by damage to vestibulo-spinal and vestibulo-cortical mechanisms, respectively. Other ocular-motor features of DBN such as its head motion/positioning sensitivity (83–86) and, in particular, the fact that the nystagmus is partly suppressed by fixation (like all vestibular nystagmus do) (81) also support a vestibular origin and argue against a pursuit/fixation mechanism.

Normal vestibular navigation in patients with DBN agrees with their clinical picture: they suffer from nystagmus-related oscillopsia and gait imbalance but do not complain of impaired navigation in the real world. These patients usually come to the clinic by public transport, are unaccompanied, and never report spatial disorientation, confusion, or cognitive problems—features never mentioned in the abundant literature on this syndrome (33, 34, 79, 87). However, SBSOD questionnaire scores were slightly reduced in those patients compared with age-matched healthy subjects, possibly due to the nystagmus-induced visual instability (oscillopsia) interfering in real-world navigation. MRI examination only finds atrophy in the vestibulo-cerebellum (flocculo-nodular lobe) or more widespread in the cerebellar vermis but nothing else of significance (23). However, it could be pointed out as a limitation in our study that normal vestibular navigation in patients with DBN may result from having only examined horizontal rotations whereas their main clinical abnormality is in the vertical plane. Nevertheless, it is well established that patients with DBN show clear abnormalities of vestibulo-ocular, optokinetic function (85, 88), and velocity-perceptual abnormalities (21) in the horizontal plane as well. We piloted experiments similar to the ones reported here but for the vertical canal system but the protocol was not well tolerated even by seasoned vestibular scientists and, thus, this remains a limitation in our study.

The vestibular navigation tasks used here are almost identical to those used in patients with congenital blindness (2) and peripheral vestibular disorders (62) but, apart from our patients with cerebellar DBN, they have not been applied to CNS disorders. Accordingly, we piloted these techniques in two patients with early dementia heralded by episodes of spatial disorientation to see if pure vestibular navigation was compromised. The prediction was that it would be because the hippocampal/para-hippocampal regions (89), temporo-parietal cortex (90), and the connectivity between those essential areas for spatial orientation, are disrupted in dementia (31, 91–93). Furthermore, these regions and associated networks contain a number of vestibularly fed cells essential for animal spatial navigation (49), such as the head direction cells that receive vestibular and visual input and encode the direction of an animal’s heading even in the dark (94). However, given that most studies on spatial orientation in cognitively impaired patients are desk based (no real motion; no vestibular stimulation), and that clinical episodes of disorientation or getting lost relate to specific visual features in the environment (69, 95–97), we just did not know if pure vestibular navigation would be affected. A series of studies reported defective otolith (but not semicircular canals) short latency reflex abnormalities in a small proportion of normal subjects with lesser cognitive function and patients with Alzheimer’s disease (98, 99) but the significance of those findings remains baffling. It is difficult to conceive how the otolith but not the canals can be associated with poor spatial cognition when studies in humans devoid of vestibular function show that it is an angular, not linear, motion that is profoundly disrupted during field navigation tasks (3). Although otolith-deficient tilted mice show dysfunctional placed cells in the hippocampus (100), mice actively navigate space vertically, unlike humans.

Our pilot results showed that the two cognitively impaired patients had poor accuracy, large variability, and frequent errors during vestibular navigation tasks. This was particularly marked during the CTC task, which could have been anticipated on the basis that this task requires a more complex central representation of external space. Although we do not wish to speculate, the findings suggest that vestibular navigation seems disrupted in dementia due to impaired central multisensory integration and that our technique is promising for future research. Also, within these restricted pilot results, it seems that the presence of degenerative cognitive impairment, despite the different underlying pathology in our two patients, is responsible for the vestibular navigation deficit. Spatial disorientation in neuro-degenerative disease is not confined to the visual world but also seems to involve vestibular-based navigation.

## DATA AVAILABILITY

Data will be made available upon reasonable request.

## GRANTS

Dr. A. Zachou was supported by scholarship granted from Hellenic Neurological Society (HNS). At the time of this work A. M. Bronstein was supported by a Research Grant R481/0516 from the Dunhill Medical Trust and a block grant from the National Institute for Health Research Imperial BRC.

## DISCLOSURES

No conflicts of interest, financial or otherwise, are declared by the authors.

## AUTHOR CONTRIBUTIONS

A.Z. and A.M.B. conceived and designed research; A.Z. performed experiments; A.Z. analyzed data; A.Z. and A.M.B. interpreted results of experiments; A.Z. prepared figures; A.Z. drafted manuscript; A.Z. and A.M.B. edited and revised manuscript; A.M.B. approved final version of manuscript.
